# Association Between Baseline Serum Lipids and Severity of Dry Eye Symptoms in Acne Patients Treated with Isotretinoin

**DOI:** 10.7759/cureus.60922

**Published:** 2024-05-23

**Authors:** Abdullah N Alajaji

**Affiliations:** 1 Department of Dermatology, College of Medicine at Qassim University, Qassim, SAU

**Keywords:** acne, lipids, cholesterol, triglycerides, dry eyes, isotretinoin

## Abstract

Background: Isotretinoin is a commonly prescribed medication for moderate-to-severe acne. Elevated triglycerides and total cholesterol, as well as eye dryness, are frequent side effects of isotretinoin.

Objective: This study aims to examine the association between serum baseline levels of triglycerides and total cholesterol with regards to the severity of dry eye symptoms in acne patients treated with isotretinoin.

Method: The study was a retrospective review of acne patients treated with isotretinoin for at least four months at the dermatology clinics of Qassim University Medical City, Saudi Arabia. Thirty patients were included in the study as they met the inclusion criteria. Baseline levels of triglycerides and total cholesterol were reviewed for these patients. The Ocular Surface Disease Index (OSDI) questionnaire was sent and filled out by the study participants to assess the severity of eye dryness.

Result: 30 patients were included in the study, with 16 (53.3%) females and 14 (46.7%) males. The average age of participants was 22.1 years. The duration of treatment was between 120 and 140 days in 13 (43.3%) participants and 140 and 180 days in 17 (56.7%) participants. The mean ± 1 standard deviation (SD) was reported for each of the three variables, with an Ocular Surface Disease Index (OSDI) score of 27.6 ± 19.2, a baseline total cholesterol of 4.4 ± 0.9 mmol/L, and a baseline triglyceride level of 0.83 ± 0.4 mmol/L. Using a multiple linear regression model, baseline triglycerides and total cholesterol were used as predictors of the OSDI score. There was a significant dependent interaction between baseline total cholesterol and triglycerides and their effect on the OSDI score, with a higher OSDI score at higher levels of both triglycerides and cholesterol and a lower OSDI score at lower levels of both triglycerides and cholesterol. The study result showed that, in acne patients treated with isotretinoin for at least four months, a higher baseline level of both triglycerides and total cholesterol is associated with worse dry eye symptoms compared to those with lower baseline levels.

Conclusion: The study concluded that baseline levels of triglycerides and total cholesterol are both significant predictors of the severity of dry eye symptoms in acne patients treated with isotretinoin. Despite study limitations due to the small sample size, we hope that, based on our findings, this will open the door to future studies with a larger sample size to further confirm our findings generalize the result, and apply it to clinical practice so that clinicians may identify those at higher risk of severe eye dryness before starting isotretinoin and subsequently be able to recommend specific measures to minimize symptoms of eye dryness.

## Introduction

Acne is a common skin condition affecting many adolescents and young adults, with an estimated prevalence of 42% to 73% among individuals less than 30 years of age [1,2]. Acne classically presents with comedones, papules, pustules, and sometimes nodules, and it mainly affects the face, upper back, and upper chest areas. Acne can be mild, moderate, or severe. Mild acne can be treated with topical medications such as topical antibiotics and topical retinoids. Moderate to severe acne is usually treated with systemic medications such as oral antibiotics or oral isotretinoin. Isotretinoin is a very effective medication for treating recalcitrant acne that has failed to respond to topical treatments and/or oral antibiotics [3]. Isotretinoin can improve acne markedly and prevent acne complications such as scarring. Isotretinoin has a direct anti-inflammatory effect through normalization of the immune response to Propionibacterium acne, and it causes shrinkage of the sebaceous glands, which causes a decrease in sebum production, thus leading to inhibition of sebum-dependent P. acne [4]. Isotretinoin side effects include teratogenicity, dry skin and mucus membranes, elevated lipids, and elevated liver enzymes [5]. Eye dryness is the most common ocular complaint among patients taking isotretinoin [6]. Other less common ocular side effects include visual changes such as decreased night vision and ocular irritation [7,8]. Lamberg et al. reviewed 41 epidemiological studies regarding isotretinoin-induced ocular side effects and found that the incidence of dry eye disease among patients taking isotretinoin was 27% [9]. Isotretinoin is known to inhibit sebum production by the meibomian gland, subsequently causing dry eyes [10,11]. Choi et al. reviewed 2272 patients to study the correlation between the prevalence of dry eye syndrome (DES) and dyslipidemia and found that men with dyslipidemia have a higher incidence of DES with an odds ratio of 1.29, after adjusting for other factors such as age, contact lens use, smoking, diabetes, and hypertension [12]. Rathnakumar et al. reviewed 60 patients with DES and 60 individuals with no DES as a control and found a significant association between dyslipidemia and DES, with a higher prevalence of DES in patients with high total cholesterol [13]. This association can be explained by the fact that elevations in serum total cholesterol and triglycerides are associated with an altered composition of lipids in the meibum, which causes an increase in sebum viscosity, blocks meibomian gland opening, and decreases meibomian gland oil production, resulting in dry eye symptoms [14]. Given the high prevalence of acne and the fact that a considerable proportion of these patients are on isotretinoin, there is a need for a better understanding of the ocular symptoms associated with isotretinoin. This study aims to investigate if there is an association between baseline triglycerides and total cholesterol and the severity of eye dryness in acne patients taking isotretinoin. The study results may give clinicians insight into acne patients who are at higher risk of severe eye dryness before starting isotretinoin. This will help clinicians identify those patients at risk based on baseline lipids, thus minimizing their risk of developing severe eye symptoms by suggesting specific measures or recommendations. 

## Materials and methods

This was a retrospective study reviewing acne patients treated with isotretinoin at Qassim University Medical City in Buraidah, Saudi Arabia, for the period between May 2022 and May 2023. Ethical approval was obtained from the Committee of Research Ethics, Deanship of Scientific Research at Qassim University on December 15, 2021, with the ethical approval number 21-05-15. We included acne patients over the age of 18 treated with isotretinoin for at least four months with a current dose of 40 mg per day at the time of the survey. Patients with a history of ocular diseases, previous eye surgery, or a history of dry eye disease before starting isotretinoin were excluded. The number of acne patients reviewed was 200, but only 107 patients responded to the survey. After excluding those patients who did not finish at least four months of treatment and those who were taking less than 40 mg of isotretinoin at the time of the survey, we ended up with a total of 30 patients who met the study criteria and replied to a questionnaire called the Ocular Surface Disease Index (OSDI), which is a validated questionnaire and has a specificity of 83% and a sensitivity of 60% in diagnosing patients with dry eye disease [15]. Patients were contacted via text message, and they filled out the questionnaire online. It is a 12-item questionnaire regarding symptoms of dry eyes, and it has three sub-items: ocular symptoms, vision-related function, and environmental triggers. All patients were taking isotretinoin when they answered the OSDI score questionnaire and the duration of treatment was between 120 and 140 days in 43.3% of them and 140 and 180 days in the remaining 56.7% of participants. Patients rated their symptoms on a 0 to 4 scale, with zero being none of the time and four being all the time (see Appendix 1 for questionnaire details). The total score ranges from 0 to 100, with scores of 12 or less classified as normal, 13 to 22 as mild dry eye disease, 23 to 32 as moderate, and more than 33 classified as severe dry eye disease [16,17]. Baseline triglycerides and total cholesterol levels were obtained from electronic medical records (EMR) for all study participants. 

Statistical analysis

Study data was analyzed using R programming language version 4.2.0. R is a statistical programming language designed for use in statistical computing. All study variables were continuous: ocular surface disease index (OSDI), baseline total cholesterol, and baseline triglycerides. They were reported as the mean and standard deviation. The functions used to identify univariate and multivariate outliers, assess normality and homogeneity of variances, and calculate summary statistics were from the Tidyverse (Wickham et al., 2019) and Rstatix (Kassambara A, 2022) packages of the R programming language [18,19]. The three variables were tested for normality using the Shapiro-Wilk test from the Rstatix package of the R system. The Spearman correlation was investigated between baseline total cholesterol and triglycerides with OSDI scores to study associations between these variables. We used functions from the Rstatix package of the R system to calculate the Spearman correlation and the significance of these correlations. A multiple linear regression was built to examine the relationship between baseline total cholesterol, baseline triglycerides, and the OSDI score. The model was fitted using the statistics from the R package (R Core Team, 2022) [20]. The residuals were checked using the DHARMa package (Hartig F, 2022) of the R system [21]. The model results were reported using the broom (Robinson D et al., 2023) and flextable (Gohel D, 2022) packages of the R system. The predicted effect of predictors on OSDI was plotted using the sjPlot package of R (Lüdecke D, 2023) to show the association between baseline triglycerides and total cholesterol and OSDI score [22-24]. A p-value < 0.05 was considered significant. 

## Results

A total of 30 patients were included in the study, and we reviewed OSDI scores, baseline total cholesterol, and baseline triglycerides. The study participants were 16 (53.3%) females and 14 (46.7%) males. The average age of participants was 22.1 years. The duration of treatment was between 120 and 140 days in 13 (43.3%) participants and 140 and 180 days in 17 (56.7%) participants. Patient demographics and characteristics are summarized in Table 1.

**Table 1:** Demographics and characteristics of study patients

Number of Patients	30
Gender	Females: 16 (53.3%) and Males: 14 (46.7%)
Age (average)	22.1 (years)
Duration of treatment among study patients	120 to 140 days in 13 (43.3%) and 140 to 180 days in 17 (56.7%)

The mean ± 1 standard deviation (SD) was reported for each of the three variables, with an Ocular Surface Disease Index (OSDI) score of 27.6 ± 19.2, a baseline total cholesterol of 4.4 ± 0.9 mmol/L, and a baseline triglyceride level of 0.83 ± 0.4 mmol/L. The mean ± standard deviation (SD) was reported for each variable in Table 2 using univariate analysis.

**Table 2:** Univariate summary statistics The data is presented as ‘mean±SD’

Variable	N = 30
Ocular Surface Disease Index (OSDI)	27.6 ± 19.2
Baseline Cholesterol	4.4 ± 0.9
Baseline Triglycerides	0.83 ± 0.4

Correlation analysis

The three variables were tested for normality using the Shapiro-Wilk test, and only the triglyceride variable was not normally distributed. Therefore, the Spearman correlation was investigated between triglycerides or cholesterol and the OSDI outcome. The Spearman correlation was found to be weak and insignificant, as shown in Table 3.

**Table 3:** Spearman correlation between cholesterol and triglycerides in relation to Ocular Surface Disease Index (OSDI) scores

Variable 1	Variable 2	Spearman correlation	p-value
Total Cholesterol	Ocular Surface Disease Index (OSDI)	-0.23	0.226
Triglycerides	Ocular Surface Disease Index (OSDI)	0.28	0.134

These findings could be due to the small sample size of the study, so we used linear regression analysis to study the association between baseline lipids and OSDI scores.

Regression analysis

A multiple linear regression model was built using baseline triglycerides and baseline total cholesterol as predictors of OSDI score. The model estimates, 95% confidence intervals, and p-values are shown in Table 4. Both triglycerides and cholesterol are significant predictors of the OSDI score. There was a significant dependent interaction between them on their effect on OSDI score, with a higher OSDI score at higher levels of both triglycerides and cholesterol and a lower OSDI score at lower levels of both triglycerides and cholesterol. This shows an association between baseline lipids and the severity of dry eye symptoms in acne patients treated with isotretinoin.

**Table 4:** Linear regression model results for Ocular Surface Disease Index (OSDI) scores

Term	Estimate (95% CI)	p-value
Intercept	130.5 (47.1-213.9)	0.0034
Total Cholesterol	-27.4 (-46.6 - -8.1)	0.0070
Triglycerides	-110.5 (-207.2 - -13.7)	0.0268
Total Cholesterol X Triglycerides	29.3 (7.7-50.9)	0.0096

**Figure 1:**
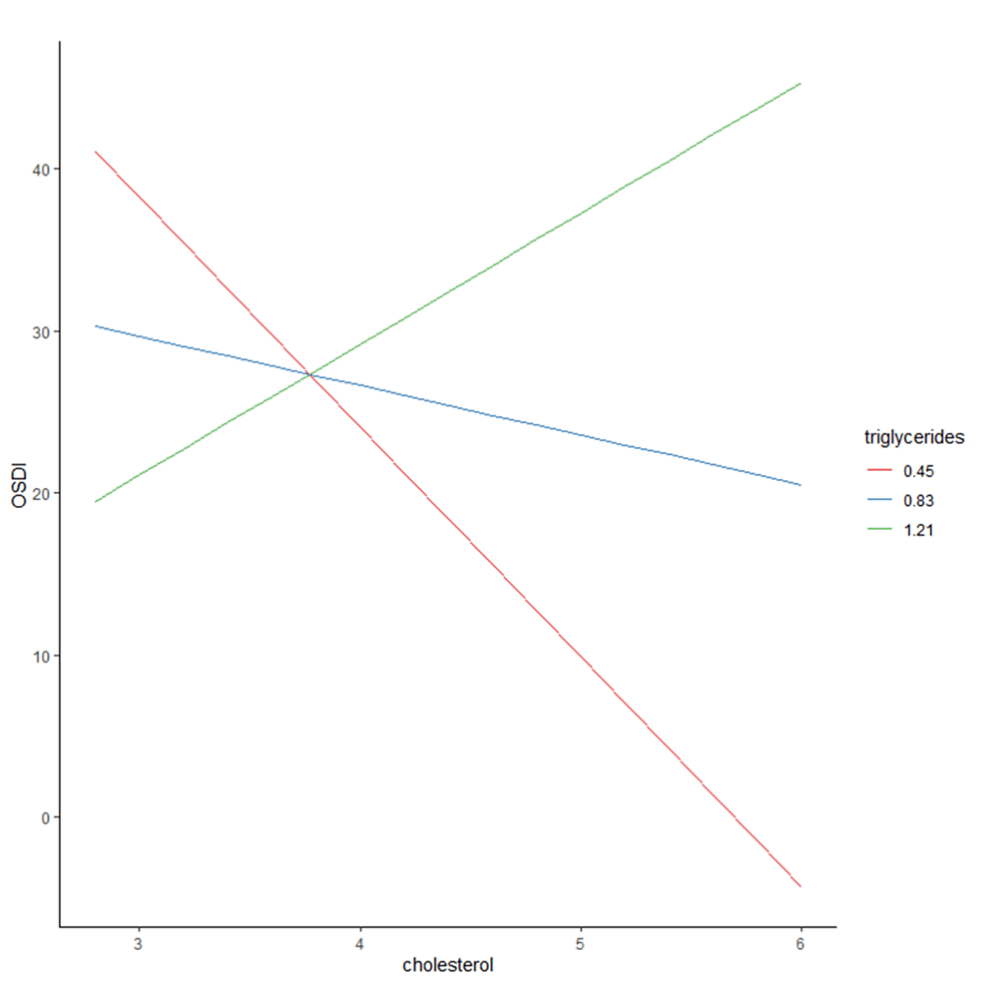
Predicted Ocular Surface Disease Index (OSDI) scores at different cholesterol levels and three triglyceride levels

To look at the effect of cholesterol on OSDI scores at different baseline levels of triglycerides, we calculated the effect of cholesterol on OSDI scores at 3 levels of triglycerides: the mean (0.83 mmol/L), the mean +1 SD (1.21 mmol/L), and the mean -1 SD (0.45 mmol/L). At 0.45 and 0.83 levels of triglycerides (low levels), elevation of the total cholesterol level was not associated with an increased OSDI score. While at a higher baseline level of triglycerides (1.21 mmol/L), a higher baseline level of cholesterol is associated with increased OSDI scores on average (Figure 1).

**Figure 2:**
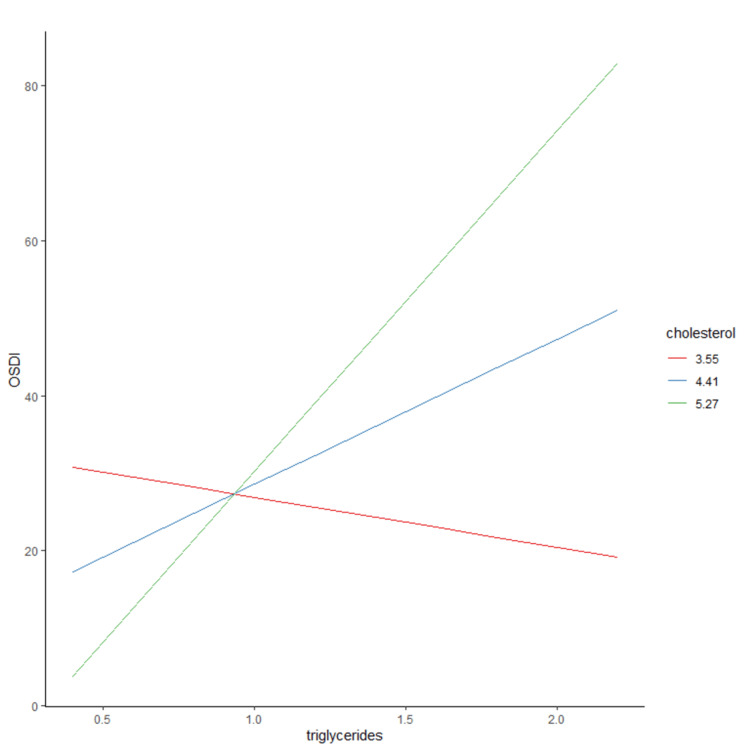
Predicted Ocular Surface Disease Index (OSDI) scores at different triglyceride levels and three cholesterol levels

Similarly, we calculated the predicted effect of triglycerides on OSDI scores at 3 levels of total cholesterol: the mean (4.41 mmol/L), the mean +1 SD (5.27 mmol/L), and the mean -1 SD (3.55 mmol/L). At low levels of cholesterol (3.55), higher triglyceride levels were not associated with an increase in OSDI score on average. While at higher levels of total cholesterol (4.41 and 5.27 mmol/L), higher baseline levels of triglycerides are associated with increased OSDI scores on average (Figure 2).

This may indicate that patients with higher baseline levels of both triglycerides and total cholesterol are more likely to have higher OSDI scores and, subsequently, are at greater risk of developing dry eye symptoms. 

## Discussion

Isotretinoin is well known to cause elevations in triglycerides and total cholesterol. Studies have shown that an increased level of total cholesterol is seen in up to 30% of patients taking isotretinoin, while hypertriglyceridemia occurs in up to 45% of these patients [25]. Several studies have shown an association between the severity of DES and dyslipidemia. In a retrospective study, Aldaas et al. reviewed 72,931 patients to study the association between DES and dyslipidemia and found that the odds of having a DES diagnosis are increased in patients with dyslipidemia [26]. In a meta-analysis, Tomoika et al. showed that high total cholesterol and high triglycerides were significantly associated with meibomian gland dysfunction and dry eyes, with odds ratios of 5.2 and 3.2, respectively [27]. Other studies showed similar results, with a higher incidence of DES in patients with dyslipidemia [28,29].

The previously mentioned studies were looking for the association between DES and dyslipidemia in patients not taking isotretinoin. Other studies mentioned above were investigating the association between isotretinoin and DES only. But, to our knowledge, studies looking at the effect of baseline lipids on the severity of DES in patients taking isotretinoin are lacking.

Given this significant association between isotretinoin and dyslipidemia and the association between the severity of dry eye symptoms and dyslipidemia, we believe that it would be helpful for clinicians to know which acne patients are at higher risk of developing DES before starting isotretinoin. In this study, we wanted to investigate if higher baseline levels of both total cholesterol and triglycerides may affect the severity of eye dryness after four months of isotretinoin treatment. It is recommended as standard care that all patients get baseline triglycerides and cholesterol before starting isotretinoin. Clinicians may stratify patients according to levels of baseline lipids and may intervene early to prevent or minimize the risk of severe dry eye symptoms.

Study limitations 

The study was limited by the small sample size, which may limit the generalizability of our study results. Other confounders that may affect the severity of eye dryness include lifestyle, other medications, smoking, environmental factors, duration of isotretinoin treatment, and variations in the frequency of applying eye lubricants among patients. Isotretinoin can affect the severity of DES, but previous research was done to examine the association between isotretinoin cumulative dose and OSDI scores and found no statistically significant association between the severity of dry eye symptoms and the total cumulative dose of isotretinoin [30]. Other limitations of the study include the retrospective nature of the study and recall bias, given that it is a questionnaire-based study. 

## Conclusions

Patients treated with isotretinoin for acne are more likely to experience dry eye symptoms as a side effect of isotretinoin. Our study showed that triglycerides and total cholesterol are both significant predictors of the severity of dry eye symptoms in these patients. We found that the severity of dry eye symptoms is increased in patients with higher baseline levels of both triglycerides and total cholesterol. Given the small sample size and other study limitations that may prevent the generalizability of our study results, we hope that this study will open the door for future studies with a larger sample size to further confirm our findings to generalize the results, and apply them to clinical practice so that clinicians treating acne patients with isotretinoin may stratify their patients based on levels of baseline lipids. This will hopefully help identify those at higher risk of developing severe eye dryness to suggest specific measures and instructions to minimize their risk of eye dryness. 

## Appendices

Appendix 1 

Ocular Surface Disease Index (OSDI) Questionnaire:

A. Have you experienced any of the following during the last week?

**Table 5:** Ocular Surface Disease Index (OSDI) Questionnaire Ocular Surface Disease Index (OSDI) score = Sum of scores for all questions answered. OSDI score categories (severity of eye dryness): 0-12: Normal, 12–22: Mild, 23-32: Moderate, > 32: Severe

Have you experienced any of the following during the last week?					
	All the time	Most of the time	Half of the time	Some of the time	None of the time
1.Eyes that are sensitive to light?	4	3	2	1	0
1.Eyes that are sensitive to light?	4	3	2	1	0
2.Eyes that feel gritty?	4	3	2	1	0
3.Painful or sore eyes?	4	3	2	1	0
4.Blurred vision?	4	3	2	1	0
5.Poor vision?	4	3	2	1	0
Have problems with your eyes limited you in performing any of the following during the last week?					
	All the time	Most of the time	Half of the time	Some of the time	None of the time
6.Reading?	4	3	2	1	0
7.Driving at night?	4	3	2	1	0
8.Working with computer or ATM?	4	3	2	1	0
Have your eyes felt uncomfortable in any of the following situations during the last week?					
	All the time	Most of the time	Half of the time	Some of the time	None of the time
10.Windy climate?	4	3	2	1	0
11.Places with low humidity (very dry)?	4	3	2	1	0
12. Areas that are air conditioned?	4	3	2	1	0

## References

[1] Collier CN, Harper JC, Cafardi JA, Cantrell WC, Wang W, Foster KW, Elewski BE (2008). The prevalence of acne in adults 20 years and older. J Am Acad Dermatol.

[2] Wolkenstein P, Machovcová A, Szepietowski JC, Tennstedt D, Veraldi S, Delarue A (2018). Acne prevalence and associations with lifestyle: a cross-sectional online survey of adolescents/young adults in 7 European countries. J Eur Acad Dermatol Venereol.

[3] Bagatin E, Costa CS (2020). The use of isotretinoin for acne - an update on optimal dosing, surveillance, and adverse effects. Expert Rev Clin Pharmacol.

[4] Dispenza MC, Wolpert EB, Gilliland KL, Dai JP, Cong Z, Nelson AM, Thiboutot DM (2012). Systemic isotretinoin therapy normalizes exaggerated TLR-2-mediated innate immune responses in acne patients. J Invest Dermatol.

[5] Brzezinski P, Borowska K, Chiriac A, Smigielski J (2017). Adverse effects of isotretinoin: A large, retrospective review. Dermatol Ther.

[6] Zakrzewska A, Wiącek MP, Słuczanowska-Głąbowska S, Safranow K, Machalińska A (2023). The effect of oral isotretinoin therapy on meibomian gland characteristics in patients with acne vulgaris. Ophthalmol Ther.

[7] Ruiz-Lozano RE, Hernández-Camarena JC, Garza-Garza LA, Bustamante-Arias A, Colorado-Zavala MF, Cardenas-de la Garza JA (2020). Isotretinoin and the eye: A review for the dermatologist. Dermatol Ther.

[8] Elubous KA, Toubasi AA, Elubous A, Alryalat SA, Abous H (2022). Ocular manifestations of systemic isotretinoin in patients with acne: a systemic review and meta-analysis. Cutan Ocul Toxicol.

[9] Lamberg O, Strome A, Jones F, Mleczek J, Jarocki A, Troost JP, Helfrich Y (2023). Ocular side effects of systemic isotretinoin - a systematic review and summary of case reports. J Dermatolog Treat.

[10] Neudorfer M, Goldshtein I, Shamai-Lubovitz O, Chodick G, Dadon Y, Shalev V (2012). Ocular adverse effects of systemic treatment with isotretinoin. Arch Dermatol.

[11] Fraunfelder FW (2004). Ocular side effects associated with isotretinoin. Drugs Today (Barc).

[12] Choi HR, Lee JH, Lee HK, Song JS, Kim HC (2020). Association between dyslipidemia and dry eye syndrome among the Korean middle-aged population. Cornea.

[13] Rathnakumar K, Ramachandran K, Baba D (2018). Prevalence of dry eye disease and its association with dyslipidemia. J Basic Clin Physiol Pharmacol.

[14] Butovich IA, Millar TJ, Ham BM (2008). Understanding and analyzing meibomian lipids--a review. Curr Eye Res.

[15] Schiffman RM, Christianson MD, Jacobsen G, Hirsch JD, Reis BL (2000). Reliability and validity of the Ocular Surface Disease Index. Arch Ophthalmol.

[16] Grubbs JR Jr, Tolleson-Rinehart S, Huynh K, Davis RM (2014). A review of quality of life measures in dry eye questionnaires. Cornea.

[17] Ozcura F, Aydin S, Helvaci MR (2007). Ocular surface disease index for the diagnosis of dry eye syndrome. Ocul Immunol Inflamm.

[18] Wickham H, Averick M, Bryan J (2019). Welcome to the Tidyverse. Jr Ope Sour Soft.

[19] Kassambara A (2022 (2024). CRAN: Rstatix: Pipe-friendly framework for basic statistical tests_. Rpackage version 0.7.1. https://CRAN.R-project.org/package=rstatix.

[20] (2024). R: A language and environment for statistical computing. R foundation for statistical computing, Vienna, Austria. https://www.R-project.org/.

[21] (2024). CRAN.Rproject: Residual diagnostics for hierarchical (multi-level/mixed) regression models. R package version 0.4.6. https://CRAN.Rproject.org/package.

[22] Robinson D, Hayes A, Couch S (2023 (2024). CRAN: Convert statistical objects into Tidy Tibbles. R package version 1.0.4. https://CRAN.R-project.org/package=broom.

[23] Gohel D (2022 (2024). CRAN: Functions for tabular reporting. R package version 0.7.0. https://CRAN.R-project.org/package=flextable.

[24] Lüdecke D (2023 (2024). CRAN: Data visualization for statistics in social science. R package version 2.8.14. https://CRAN.R-project.org/package=sjPlot.

[25] Zane LT, Leyden WA, Marqueling AL, Manos MM (2006). A population-based analysis of laboratory abnormalities during isotretinoin therapy for acne vulgaris. Arch Dermatol.

[26] Aldaas KM, Ismail OM, Hakim J, Van Buren ED, Lin FC, Hardin JS, Meyer JJ (2020). Association of dry eye disease with dyslipidemia and statin use. Am J Ophthalmol.

[27] Tomioka Y, Kitazawa K, Yamashita Y (2023). Dyslipidemia exacerbates meibomian gland dysfunction: a systematic review and meta-analysis. J Clin Med.

[28] Dao AH, Spindle JD, Harp BA, Jacob A, Chuang AZ, Yee RW (2010). Association of dyslipidemia in moderate to severe meibomian gland dysfunction. Am J Ophthalmol.

[29] Irfan KS, Agrawal A, Singh A, Mittal SK, Samanta R, Shrinkhal Shrinkhal (2020). Association of lipid profile with severity of meibomian gland dysfunction. Nepal J Ophthalmol.

[30] Alfouzan YA, Al-Hammad RA, Alkhuzayem FA, Alkhudair RF, Alotaibi MA, Alajaji AN, Al-Muhaylib AA (2023). Isotretinoin-related eye dryness in acne patients in Qassim, Saudi Arabia. Cureus.

